# Aggregation of Aß(25-35) on DOPC and DOPC/DHA Bilayers: An Atomic Force Microscopy Study

**DOI:** 10.1371/journal.pone.0115780

**Published:** 2014-12-31

**Authors:** Matilde Sublimi Saponetti, Manuela Grimaldi, Mario Scrima, Cristiano Albonetti, Stefania Lucia Nori, Annamaria Cucolo, Fabrizio Bobba, Anna Maria D'Ursi

**Affiliations:** 1 Physics Department and Research Centre for Nanomaterials and Nanotechnology, University of Salerno, Via Giovanni Paolo II, 132, 84084, Fisciano (SA), Italy; 2 Institute for Superconductivity and Innovative Materials of the Italian National Research Council, Via Giovanni Paolo II, 132, 84084, Fisciano (SA), Italy; 3 Department of Pharmacy, University of Salerno, Via Giovanni Paolo II, 132, 84084, Fisciano (SA), Italy; 4 Department of Medicine and Surgery, University of Salerno, Via Salvatore Allende, 84081, Baronissi (SA), Italy; 5 Institute Study Nanostructurated Materials of the Italian National Research Council, Via P. Gobetti 101, 40129, Bologna, Italy; University of Bologna & Italian Institute of Technology, Italy

## Abstract

β amyloid peptide plays an important role in both the manifestation and progression of Alzheimer disease. It has a tendency to aggregate, forming low-molecular weight soluble oligomers, higher-molecular weight protofibrillar oligomers and insoluble fibrils. The relative importance of these single oligomeric-polymeric species, in relation to the morbidity of the disease, is currently being debated. Here we present an Atomic Force Microscopy (AFM) study of Aβ(25–35) aggregation on hydrophobic dioleoylphosphatidylcholine (DOPC) and DOPC/docosahexaenoic 22∶6 acid (DHA) lipid bilayers. Aβ(25–35) is the smallest fragment retaining the biological activity of the full-length peptide, whereas DOPC and DOPC/DHA lipid bilayers were selected as models of cell-membrane environments characterized by different fluidity. Our results provide evidence that in hydrophobic DOPC and DOPC/DHA lipid bilayers, Aβ(25-35) forms layered aggregates composed of mainly annular structures. The mutual interaction between annular structures and lipid surfaces end-results into a membrane solubilization. The presence of DHA as a membrane-fluidizing agent is essential to protect the membrane from damage caused by interactions with peptide aggregates; to reduces the bilayer defects where the delipidation process starts.

## Introduction

β-amyloid plaques composed of β-sheet-rich fibrillar aggregates of peptides Aβ(1-40) and Aβ(1-42), are the hallmark of the brain in Alzheimer disease (AD) patients. Aβ peptides are produced in the form of soluble molecules but, in response to environmental factors, they aggregate into low-molecular-weight soluble oligomers and higher-molecular-weight protofibrillar oligomers (PFOs), which in turn give rise to insoluble fibrils, forming amyloid plaques [1]–[3].

Although insoluble fibrils have long been considered as being responsible for the disease, the correlation between the presence of insoluble fibrillar deposits and the manifestation of the disease is currently under debate. Conversely soluble Aβ oligomers, ranging in size from dimers up to particles of one million daltons or larger prove to correlate better with dementia [4]–[7], suggesting that oligomeric forms of Aβ may be the primary toxic species. Soluble oligomers are recognized as the primary toxic species in AD, as well as in many other degenerative diseases, whereas the accumulation of large fibrillar deposits is considered either inert, even protective, or pathological but by different mechanisms [8]–[11].

In the early stages of the aggregation process, Aβ(1-40) and Aβ(1-42) PFOs appear as globular aggregates with diameter ranging from 3 to 10 nm, as shown by Atomic Force Microscopy (AFM) images. PFOs evolve into fibrils and plaques at a later time or, may combine to form curved strings of globular aggregates, ring- or pore-like structures, generally referred to as annular protofibrils (APFs). [7], [12]–[13]

The toxicity of amyloidogenic Aβ protein is significantly related to its ability to interact with neuronal cell membranes: the membrane surface may be catalytic due to misfolding and aggregation of amyloidogenic proteins, and conversely amyloidogenic proteins can affect membrane structure and functionality in a still-debated mechanism that may involve membrane thinning, permeabilization, [14]–[15] and delipidation. The composition and physiochemical state of the cell membrane can regulate the size and shape of Aβ aggregates as well as their aggregation kinetic.

The highly unsaturated omega-3 fatty acids docosahexaenoic 22∶6 (DHA) and eicosapentaenoic 20∶5 (EPA) confer hyper-fluidizing properties to the membrane and enhance crucial membrane processes [16]. These compounds are found to be decreased in AD brain tissue [15], suggesting a neuroprotective role [17]. Nowadays, omega-3 fatty acids are increasingly being proposed as dietary supplements that may reduce the risk of disease development or progression [18]–[20].

The fragment including the 25–35 residues of Aβ(1–42), namely Aβ(25–35) [21], is the shortest fragment of the Aβ(1–42) peptide that remains biologically active. When aggregated, it exhibits large β-sheet structures, retaining the same physical, biological and toxicological properties as the full-length peptide. Accordingly, Aβ(25–35) has been extensively investigated as a model peptide because its short length readily allows derivatives to be synthesized [21]–[23].

We previously carried out NMR studies of the synthetic β-amyloid fragments Aβ(25–35) in different membrane-mimicking conditions, revealing the impact of specific membrane composition on the conformational state of soluble amyloid peptides [24]–[26]. Moreover, using EPR spectroscopy, we analyzed the effect of Aβ(25–35) on several membrane-mimicking bilayers characterized by different composition, and in the presence of several different bioactive compounds [27]–[35].

This work aims to visualize the Aβ(25–35) aggregates on lipid bilayers with and without DHA by AFM performed in buffer solution, *in situ* and in quasi real time. Aβ(25-35) aggregation was investigated on three surfaces: i) freshly-cleaved mica; ii) dioleoylphosphatidylcholine (DOPC) bilayer; and iii) DOPC/DHA bilayer. The mica was used as a reference surface for Aβ(25-35) aggregation, whereas DOPC and DOPC/DHA were selected as model bilayers mimicking the chemical/physical properties of the membrane environment. Our results provide evidence that in hydrophobic DOPC and DOPC/DHA lipid bilayers, Aβ(25-35) forms layered aggregates composed of mainly annular structures. The mutual interaction between annular structures and lipid surfaces end-results into a membrane solubilization. The presence of DHA as a membrane-fluidizing agent is essential to protect the membrane from damage caused by interactions with peptide aggregates; to reduces the bilayer defects where the delipidation process starts.

## Materials and Methods

### Materials

Dichloromethane and methanol, HPLC-grade solvents, were purchased from Merck (Darmstadt, Germany), while 1,1,1,3,3,3-hexafluoroisopropanol (HFIP) was supplied by Sigma-Aldrich (St. Louis, MO, USA). 1,2-dioleoyl-sn-glycero-3-phosphocholine (DOPC) and 1,2-didocosahexaenoyl-sn-glycero-3 phosphocholine (22∶6(cis)PC) were obtained from Avanti Polar Lipids (Birmingham, AL, USA).

### Peptide synthesis

The Aβ(25–35) peptide, GSNKGAIIGLM, was manually synthesized by conventional solid-phase chemistry using the Fmoc/tBu strategy and subsequently purified as previously reported (27) The peptide was characterized on a Finnigan LCQ Deca ion trap instrument equipped with an electrospray source (LCQ Deca Finnigan, San José, CA, USA). The samples were directly infused into the ESI source using a syringe pump at a flow rate of 5 µl/min. Data were analyzed with Xcalibur software. Sample purity was >98%.

In order to ensure sample reproducibility and removal of aggregated states, dry peptide was pretreated with neat TFA (from Fluka; St. Louis, MO, USA) for 3 h, followed by 10-fold dilution with MilliQ water and lyophilization (Millipore, Billerica, MA, USA).

### Lipid bilayer preparation

The DOPC molecule and the molecular mixture DOPC/DHA (in a ratio of 4∶1) were dissolved in chloroform solutions, dried by means of nitrogen gas flow and, lastly, placed in a vacuum drier for 8 h in order to remove traces of solvent. Then, DOPC and DOPC/DHA were hydrated with a Sodium phosphate buffer solution (PBS) pH = 7.4 until a final concentration of 1 mg ml^−1^ was reached. Both solutions were stirred at room temperature for dispersing the lipid suspensions and tip-sonicated for 45 minutes to obtain small unilamellar vesicles (SUVs) ranging from 40 to 50 nm [36]. To prepare the lipid bilayers, 100 µl of DOPC and DOPC/DHA SUVs solutions were drop-casted on freshly-cleaved high-grade V-1 muscovite mica discs previously glued onto a Teflon disc. After 1 hour at room temperature, the vesicle fusion process which occurs at the mica surface forms ordered and compact lipid bilayer films [37]. Therefore, the residual SUVs solutions were gradually replaced with fresh buffer in order to remove the excess lipid material and keep DOPC and DOPC/DHA bilayers fully wetted. In order to optimize the mica surface coverage (Θ), 20 bilayers were prepared and the lipid bilayer stability was checked by a continuous AFM imaging of the same area for about 3 hours.

### Peptide deposition

For experiments under dry conditions, Aβ(25-35) was dissolved in PBS (pH = 7.4) until a concentration of 200 µM and incubated at 37°C for 7 hours. At regular time intervals (1 hour), AFM samples were prepared by dropping 10 µl of the Aβ(25-35) solution onto a freshly-cleaved mica surface. After 5 minutes, samples were rinsed twice with ultrapure water, dried with nitrogen and transferred to the AFM microscope for imaging [38].

For experiments under liquid conditions, Aβ(25-35) was dissolved in PBS (pH = 7.4) up to a concentration of 20 µM, then 100 µl of the peptide solution was injected into the AFM fluid cell. Within the fluid cell, the peptide solution evaporates less than when exposed to the air, allowing up to 6 hours of continuous measurements. As a rule of thumb, the aggregation process finishes when the system reaches equilibrium, *viz.* aggregates saturate in size and shape. We performed four experiments on mica surface and ten experiments on lipid bilayers.

### Atomic Force Microscopy measurements

AFM measurements were performed using a Multimode Nanoscope V system (VEECO, Santa Barbara, CA), equipped with a 15 µm scanner (EVLR-scanner), in tapping mode configuration.

For imaging under dry conditions, antimony (n) doped Si cantilevers with a spring constant of about 30N/m and a nominal tip radius of 10 nm (NCHV VEECO) were used. The cantilever resonant frequency was about 284 kHz with a quality factor Q of 254. The scan rate was 2 Hz.

For experiments under liquid conditions, the AFM microscope used a fluid cell with an O-ring seal. AFM imaging was performed in tapping mode using silicon nitride cantilevers with a spring constant of 0.2 N/m (DNP-S VEECO) and a nominal tip radius of 10 nm. In liquid, the cantilever resonant frequency was about 7 kHz, with a quality factor Q of 20. The scan rate ranged from 1 to 2 Hz. All in-liquid AFM measurements were performed in PBS solution, in quasi real time and *in situ*. The time evolution of the Aβ(25-35) aggregation was followed for 6 hours (at most) with time-steps of 30 minutes in the first 2 hours and every hour for the remaining 4 hours. Each AFM image takes up to 17 minutes, so experiments are *de facto* in quasi real time. They are also *in situ* because the same surface area is investigated over time. The aggregation process was monitored until saturation, i.e. when the Aβ(25-35) aggregates were stable in size and shape *vs.* time. Aβ(25-35) aggregation on the mica surface was monitored with an average tip-sample force <F_ts_> of 55pN, whereas peptide aggregation on lipid bilayers was monitored with an <F_ts_> well below the rupture force of the bilayer (about 300 pN for DOPC and 150 pN for DOPC/DHA) to avoid any damage induced by the movement of the tip. Changes in <F_ts_> during experiments are reported in the Results section. Experiments were performed in a glove-box in order to keep the temperature constant (over one hour it oscillates by less than 0.3°C) and to reduce any acoustic noise interferences.

The arithmetic average of the absolute values of heights and depths from the height mean value of the image, commonly called Ra parameter, has been used to measure the surface roughness of images and it is indicated across the manuscript with the symbol σ.

## Results

### Aggregation of Aβ(25-35)

**Figure 1:**
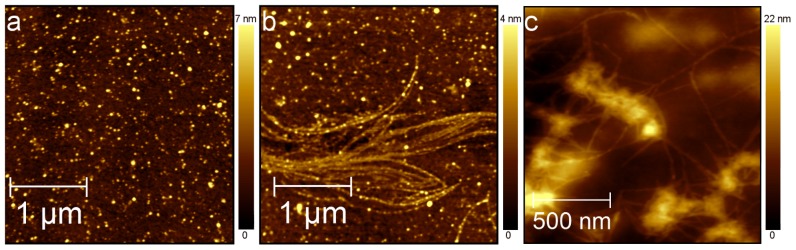
Aggregation of Aβ(25-35). Topographic AFM images of Aβ(25-35) aggregates grown in PBS at 200 µM and deposited on mica. AFM measurements were performed in tapping mode and in air. (a) After 1 hour Aβ(25-35) is organized into dispersed spherical aggregates (3×3 µm^2^, 615×615px2); (b) after 5 hours into beaded protofibrils (3×3 µm^2^, 615×615px2); (c) after 7 hours into mature fibrils (1.5×1.5 µm^2^, 307×307px^2^).

As a reference experiment, the time evolution of Aβ(25-35) aggregates grown in PBS at 200 µM was monitored by AFM measurements of dry samples prepared at successive times throughout the aggregation process. After 1 hour of PBS incubation, the Aβ(25-35) solution deposited on mica surface showed globular aggregates characterized by heights ranging from 2 to 13 nm (Fig. 1a). Four hours later, globular aggregates and linear protofibrils with lengths ranging from 0.5 to 5 µm were observed (Fig. 1b). Seven hours later, protofibrils had evolved into mature fibrils similar to those extensively described for the full-length peptide Aβ(1-42) [39] (Fig. 1c).

To investigate the evolution of Aβ(25-35) in real time and under liquid conditions, the concentration of the peptide solution was reduced to 20 µM. This concentration was the best compromise to increase aggregation time (so as to slow down aggregation rate) and to have stable conditions for AFM imaging. These AFM experiments were carried out on three different surfaces: one hydrophilic (mica) and two hydrophobic (DOPC and DOPC/DHA).

### Aggregation of Aβ(25-35) on mica

On freshly-cleaved mica, Aβ(25-35) aggregation was monitored by adding 100 µl of peptide solution into the fluid cell, while AFM scans continuously for about 6 hours. From the beginning of the deposition, Aβ(25-35) forms a smooth homogeneous layer which increased the roughness σ of the bare mica surface from 0.06 nm to 0.11 nm.

**Figure 2:**
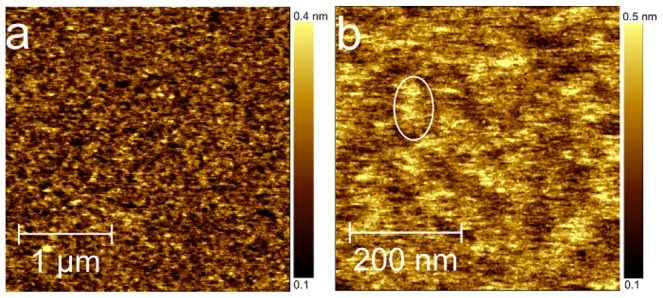
Aggregation of Aβ(25-35) on mica. Topographic AFM images of Aβ(25-35) aggregates grown on mica. Images were acquired in tapping mode and in PBS. (a) After 6 hours, Aβ(25-35) forms a smooth homogeneous layer without any well-formed aggregates (3×3 µm^2^, 2459×2459px^2^). (b) On a smaller scale the peptide tends to organize into a texture with some discernible protrusions (white ellipse) (500×500 nm^2^, 1228×1228px^2^).

After 6 hours (Fig. 2a), we did not observe any Aβ(25-35) well-formed aggregates similar to the ones discussed for Fig. 1, though on a smaller scale, (Fig. 2b) a texturing driven by protrusions such as the one highlighted by the white ellipse can be discerned. By Power Spectral Density Function (PSDF) analysis, the average protrusion size was deduced to be 50 nm [40].

### Aggregation of Aβ(25-35) on the DOPC bilayer

The DOPC bilayer was formed at room temperature on freshly-cleaved mica by the previously described vesicle fusion method. Before Aβ(25-35) deposition, DOPC morphology was investigated in buffer solution by AFM to check mica surface coverage and lipid bilayer stability during imaging. In order to optimize the lipid coverage (Θ_DOPC_) of the mica surface, lipid bilayers were prepared several times (see Material and Methods) obtaining a maximum coverage of 96% and an average coverage of (92±4)%.

**Figure 3:**
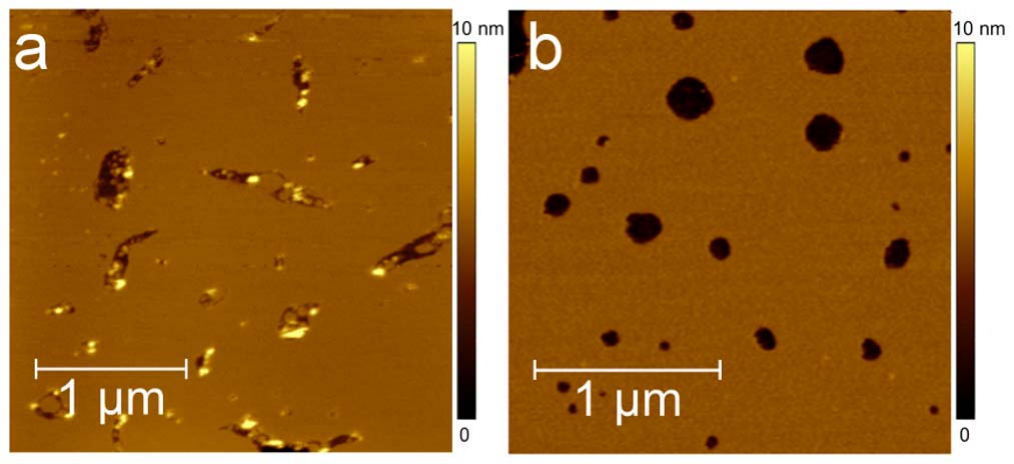
DOPC and DOPC/DHA lipid bilayers. (a) Height AFM images of DOPC (2.5×2.5 µm^2^ 1024×1024px^2^) and DOPC/DHA (b) (2.5×2.5 512×512px^2^) lipid bilayers performed in tapping mode and in PBS. The bilayers cover 92% (DOPC) and 96% (DOPC/DHA) of the mica surface. On DOPC lipid particles collected at the border or within defects are due to incomplete bilayer formation or to defective rinsing.

Fig. 3a shows a DOPC bilayer covering 92% of the mica surface. Defects characterized by different shapes and sizes expose the remaining 8% of the underlying mica surface. Some lipid particles with an average diameter of 70±7 nm and height 5±1 nm, as evaluated by measuring the full width at half maximum (FWHM) of ten particle profiles, are collected at the border or within defects. The disrupted lipid areas were characterized with the respective profile line analysis showing a thickness of 4.5±0.5 nm, which is consistent with the known thickness of a lipid bilayer. Following the experimental protocol reported by Atwood et al. [41], DOPC bilayer thickness was also more carefully measured by indentation curve analysis (Figure S1 in S1 File). Among all the acquired approaching curves only one single jump of rupture occurred, consistently with the presence of a single bilayer film. From the statistical analysis, the rupture force F_B_ = 6.8±1.1nN (Figure S2 in S1 File) and the thickness Z_B-C_ = 4.3±0.3 nm (Figure S3 in S1 File) were deduced. DOPC stability was verified by repeating AFM scans on the same area over about 3 hours. To avoid damage to the sample or the tip, the tip-sample force <F_ts_> applied during scans was about 300pN, well below the rupture force of the bilayer.

**Figure 4:**
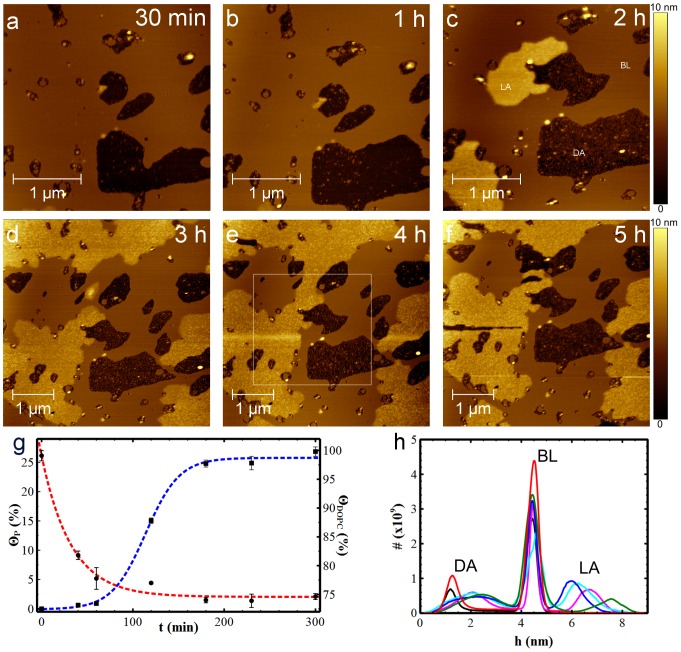
Aβ(25-35) aggregation on DOPC lipid bilayer. 3×3 µm2 (a-c), and 5×5 µm2 (d-f), topographic AFM images (1024×1024px^2^) showing the time evolution of Aβ(25-35) on DOPC lipid bilayer. AFM measurements were performed in tapping mode and in PBS with a continuous scanning of the same area after the addition of the peptide. Over time, Aβ(25-35) forms layered aggregates (LA) on the bilayer surface, bilayer defect areas increase in size and Aβ(25-35) gradually covers the disrupted areas with disordered aggregates (DA). (g) Graph showing the increase in surface area covered by LA (blue line) and the evolution of lipid bilayer over time (red line). Data were qualitatively fitted by rational and sigmoidal functions for the DOPC bilayer (θDOPC) and the LA domains (θP), respectively. Fitting curves act as a guide-to-the-eye. (h) Height distribution histograms measured at t = 30 min (black), 1 h (red), 2 h (green), 3 h (blue), 4 h (cyan) and 5 h (magenta). The squared dashed-line in (e) indicates the area where statistical analyses on 5×5 µm2 AFM images were performed so as to keep the same scan size amongst images.

Aβ(25-35) aggregation on the DOPC bilayer was monitored by injecting Aβ(25-35) previously dissolved in PBS into the AFM fluid cell. This was considered to be the starting time, t = 0, after which continuous AFM scans were carried out at different sampling times. Fig. 4a-4f show Aβ(25-35) on the DOPC bilayer at 30 min, 1 h, 2 h, 3 h, 4 h and 5 h respectively. After 30 minutes (Fig. 4a), the bilayer defects observable even in the absence of the peptide grew to 10%. At the same time, two different peptide aggregates appeared: i) layered aggregates (LA) starting from the edges of the disrupted areas and growing as an additional film on the lipid surface; and ii) disordered aggregates (DA) deposited on the mica surface of the disrupted areas.

Inspection of Fig. 4a-f shows the time evolution of the dimension of both lipid film and LA domains. This evolution is quantitatively evaluable in the graph in Fig. 4g. The DOPC bilayer has a variable surface coverage, Θ_DOPC,_ from a starting value of 92% at t = 0 min to 76% at t = 100 min and, lastly, to 75% at t = 5 h. On the other hand, LA coverage increases slowly during the first hour, from 0.5% to 0.7% (Fig. 4a and 4b), and enlarges faster from 0.7% to 26% during the following two hours, with a linear rate of 0.2%/min (Fig. 4d). Finally, LA domains reach a plateau at t = 3 h, when equilibrium between adsorbed and desorbed peptides is reached.

Analysis of DA aggregates indicates that they essentially consist of a smooth layer of peptide showing a roughness σ_DA_ = 0.35 nm at t = 30 min [42] and evolving to σ_DA_ = 0.73 nm at t = 180 min.

During scanning we did not observe any accumulation of material at the edge of the image area as a consequence of tip scan motion and this was verified after enlarging the scanned area from 3×3 µm^2^ to 5×5 µm^2^.

The time evolution of LA and DA thicknesses can be measured by the height distribution plot in Fig. 4h, as deduced by a statistical analysis performed on the common subarea indicated by the squared dashed line in Fig. 4e. Moreover, to compare the histograms at different times, the bilayer surface, which is the most statistically relevant surface in AFM images, was taken as reference height. Consequently, the histograms at the different times were adjusted to fit the BL peaks at the value h_BL_ = 4.5 nm. Over time, the DA peak moves towards the BL peak, reducing their reciprocal separation from 2.9±0.3 nm to 2.3±0.2 nm, corresponding to thicknesses of the peptide layer increasing from h_DA_(30 min) = 1.6±0.7 nm to h_DA_(5 h) = 2.2±1.0 nm. In addition, the DA peak broadens from 0.4 nm at the initial time to about 1 nm for the subsequent times, as measured by FWHM, suggesting a wider size distribution of the peptide aggregates formed above the mica surface. Regarding the LA aggregates, they become visible after two hours, having an average thickness h_LA_(2 h) = 3.1±0.9 nm, as calculated by the separation between the LA peak and BL peak. The thickness decreases over time to the value h_LA_(3 h–4 h) = 1.6±0.7 nm and finally reaches the value h_LA_(5 h) = 2.2±0.7 nm. This decrease in LA domain thickness is consistent with re-organization phenomena similar to those observed in organic ultra-thin films [43].

### Aggregation of Aβ(25–35) on DOPC/DHA bilayer

The time evolution of Aβ(25–35) aggregation was also studied on DOPC bilayers enriched with 20% DHA. Before Aβ(25-35) deposition, the morphology of the DOPC/DHA bilayer was analyzed in buffer solution by AFM. To optimize lipid bilayer coverage (Θ_DOPC/DHA_) of the mica surface, the DOPC/DHA bilayer was prepared several times (see Material and Methods), obtaining an average surface coverage of 96±2%, with a maximum extent of 99%. Fig. 3b shows the DOPC/DHA bilayer before Aβ(25–35) addition. The bilayer covers 96% of the mica surface with sparse defects on the remaining 4%. Analysis of indentation curves as previously described for DOPC indicates a rupture force F_B_ = 4.6±0.4nN and a thickness Z_B-C_ = 4.1±0.1 nm (see S1 File). The stability of the bilayer was verified by scanning the same area for about 3 hours using an <F_ts_> of about 150pN. This force, well below the rupture force of the bilayer, did not induce any damage in the bilayer due to tip movement.

We note that the addition of DHA to the DOPC bilayer reduces the rupture force but does not consistently modify bilayer thickness. The different rupture force values between DOPC and DOPC/DHA entail a change in the mechanical response of the bilayer correlated to the different lipid composition.

**Figure 5:**
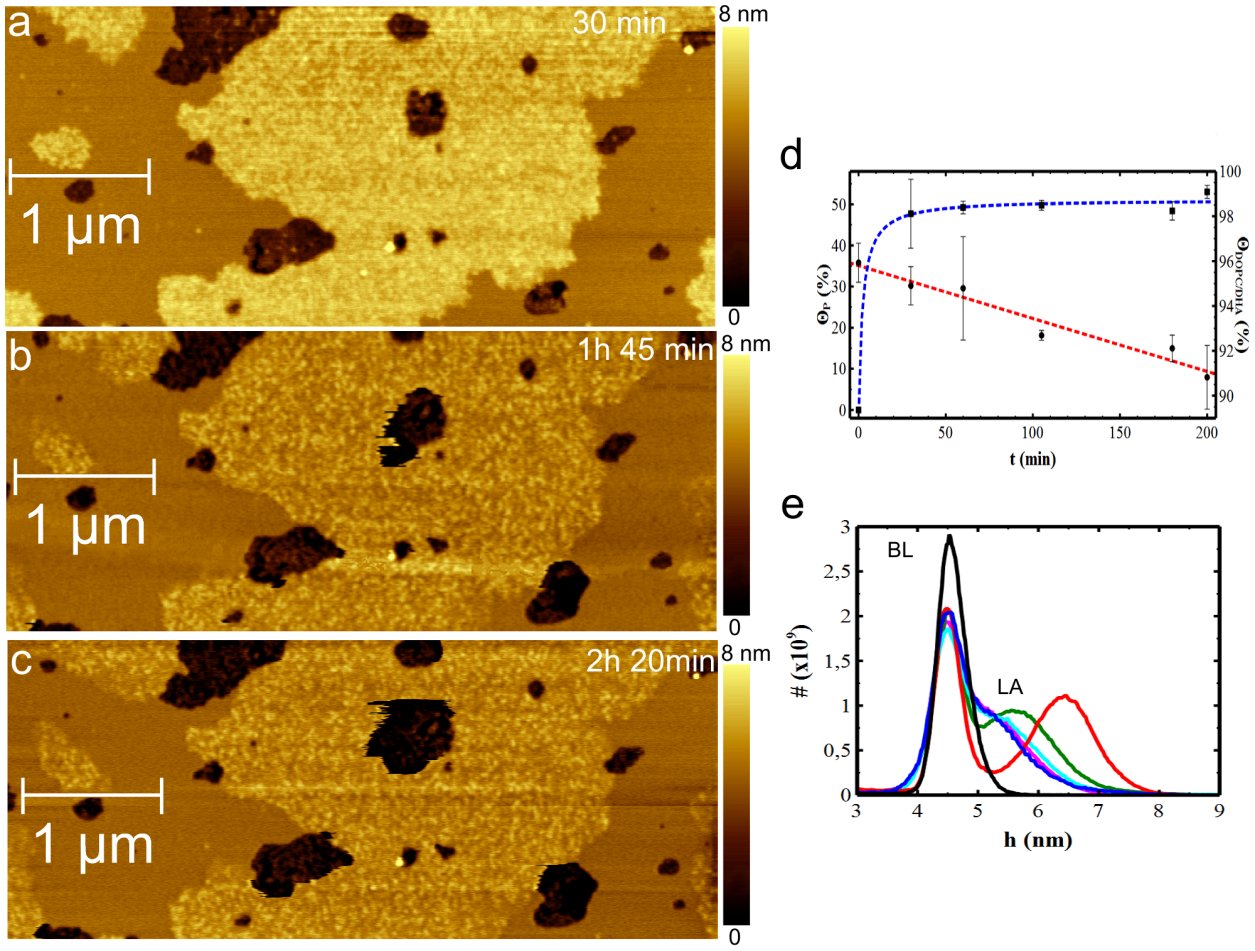
Aβ(25-35) aggregation on DOPC/DHA lipid bilayer. (a-c) 5×2.2 µm^2^ (1024×431px^2^) topographic AFM images of Aβ(25-35) aggregation on DOPC/DHA lipid bilayers. (d) Graph showing the increasing surface area covered by LA (blue line) and the lipid bilayer evolution over time (red line). LA structures were grown within the first 30 min of peptide deposition while the lipid surface area decreased slowly from 96% to 91%. Data were qualitatively fitted by rational and sigmoidal functions for the DOPC/DHA bilayer (Θ_DOPC/DHA_) and the LA domains (Θ_P_), respectively. Fitting curves act as guide-to-the-eye. (e) Height distribution histograms measured at t = 0 min (black), 30 min (red), 1 h (green), 1 h45 min (blue), 2 h (cyan) and 2 h20 min (magenta).

In order to monitor Aβ(25-35) aggregation on the DOPC/DHA bilayer, the peptide previously dissolved in PBS was injected into the AFM fluid cell. This time was considered to be the starting time (t = 0). Continuous AFM scans were carried out on an area of 3×3 µm^2^ and the growth of Aβ(25-35) aggregates was monitored with sampling times of 30 min, 1 h, 1 h45 min, 2 h and 2 h20 min (Fig. 5a-c shows representative images for 30 min, 1 h45 min and 2 h20 min). Even in the case of DOPC/DHA the peptide expands as a layer above the bilayer surface. Fig. 5d shows the evolution of coverage for both lipid film and LA domains. Θ_DOPC/DHA_ decreases from 96% (t = 0) to 91% (t = 2 h20 min). LA domains slowly increase in size, reaching an almost stable dimension (49%) after 1 hour, and 51% at the end time (t = 2 h20 min).

Comparison of Aβ(25-35) aggregation on DOPC and on DOPC/DHA bilayers indicates that in the first 30 minutes LA domains grow faster on DOPC/DHA than on DOPC and, in addition the DOPC/DHA bilayer's propensity to delipidate appears to be lower than in the case of DOPC.

As 30 min is a time interval comparable to the time needed to scan one image, the previous growth steps of LA domains on DOPC/DHA bilayer are not measurable under our experimental conditions.

Fig. 5e shows an overlapping of the plots relative to the height distributions for DOPC/DHA (BL) and peptide aggregates extending above the bilayer (LA). Again, the height histograms corresponding to each scanning time are fitted at the level of the BL peak that was set at the value of 4.5 nm. The DA peak is statistically negligible, hence it was not considered in the plot. At t = 30 min, the LA peak is already visible, measuring a thickness of h_LA_(30 min) = 1.9±0.7 nm. After 1 hour, LA thickness appears to have reduced to h_LA_(1 h) = 1.1±0.8 nm while at t = 1 h 45 min, the LA peak almost overlaps the BL peak, indicating a thickness of about 0.7 nm which is maintained until the experiments end at 2 h20 min.

Similarly to the case of DOPC, LA thickness initially increases but then falls, suggesting peptide re-organization in the peptide/lipid system.

### LA domains

**Figure 6:**
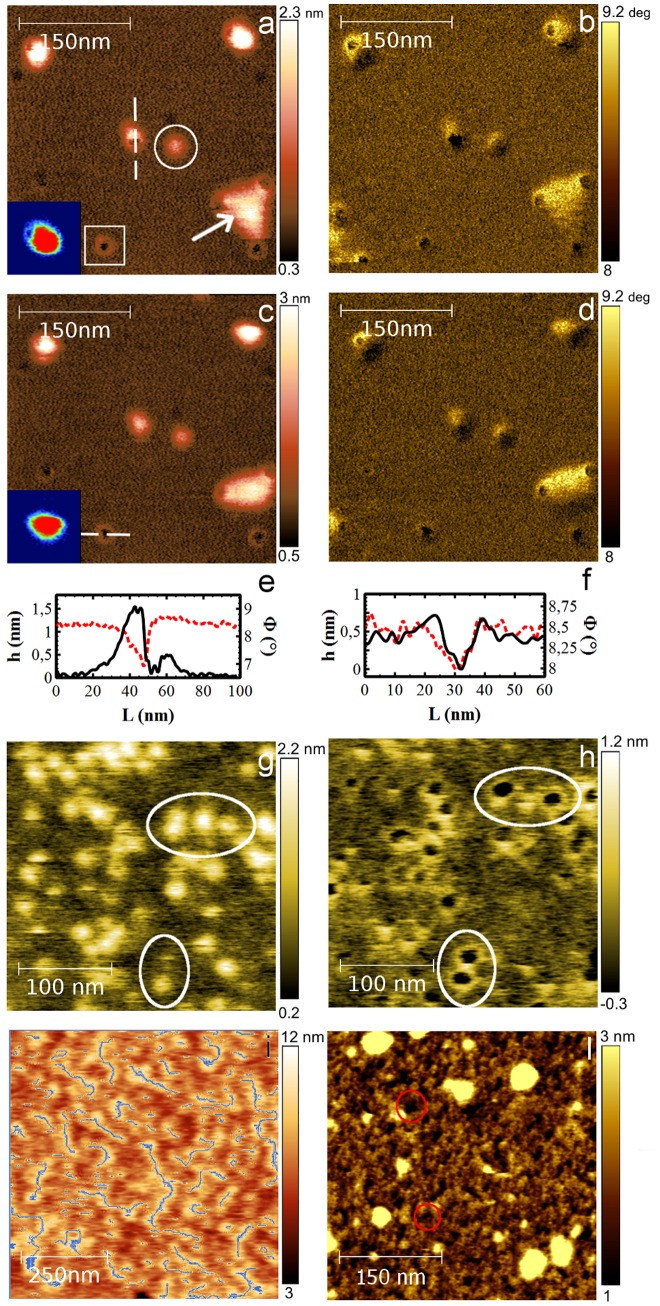
Aβ(25-35) high resolution analysis. Topographic AFM images (a, c) and corresponding phase images (b, d) performed in PBS on the same area (360×360 nm^2^, 1024×1024 px^2^) of Aβ(25-35) LA domains on the DOPC bilayer. Images were acquired with an average tip-sample force of 170pN by scanning from left to right (a, b) and from right to left (c, d). Topographies clearly show both globular aggregates (circled) and annular structures (squared). The complex LA aggregate (arrow) in the bottom right-hand corner and the globular aggregate in the top right-hand corner (insets) are perturbed by the tip movement. (e, f) Height (black) and phase (red) line profiles of globular (e) and annular structures (f) measured along the white dashed line in Fig. 6a and 6c. (g,h) Height images of a highly dense globular structure region (286×286 nm^2^, 574×574px^2^) acquired with two different tip-sample forces. At 220 pN (g), the globular structures are not perturbed, while at 234 pN (h) they are mechanically removed leaving the underlying annular structures. (i, l) AFM height images (770×770 nm^2^, 633×633 px^2^) of LA on two different regions of the DOPC/DHA bilayer. After 1 h45 min of peptide deposition (i) the LA presents a linear organization highlighted by grey fibres. After 2 h20 min (l) the LA forms a structured layer where linear organization is less visible though still distinguishable. In some locations, it is organized into annular structures (red circles) similar in dimension but more sporadic and of different nature compared to the ones observed on DOPC.

In order to understand the morphology of LA domains, high-resolution AFM images were acquired on a small scan area of 360 nm×360 nm (Fig. 6a) by scanning the surface with an average tip-sample force of 170pN. Under these conditions, as is evident in Fig. 6a, distinct peptide aggregates are visible, having globular (circled in the Figure) or annular (squared) shapes; in addition, complex LA domains are also observable at the bottom right-hand corner of the scanned area (labeled by an arrow). Globules have a diameter of about 40 nm, protruding in average 3 nm above the lipid surface. In order to fully characterize these aggregates, and thus avoid any inaccuracies deriving from experimental artifacts, we repeated the image scanning by varying the experimental parameters and acquiring topological and phase angle profiles.

Fig. 6a and Fig. 6c correspond to two images of the same area scanned from right to left (Fig. 6a) and from left to right (Fig. 6c). By comparing Fig. 6a and 6c, it is evident that the globular aggregates are present in both images but, as a consequence of the interaction with the tip, they appear to have shifted away from the centre of the rings, in the same direction as the tip scanning (see the cropped image insets in Fig. 6a, c).

Fig. 6e and 6f show the topographic and phase profiles measured across globular and annular aggregates respectively (white dashed line in Fig. 6a, 6c). The height profile indicates that the globule is about 1.5 nm in height, whereas the annular corona protrudes about 0.2nm from the lipid surface. Note that, across the whole profile, the phase angle remains positive throughout, ranging between 6°–10° respect to the 0° phase angle value centred at the resonance frequency. This means that by operating AFM in tapping mode with this chosen set-point amplitude, tip-sample interaction is kept always attractive both across the profile line and across the whole image (Fig. 6b, 6d) [44]. This rules out the possibility that the appearance of these linked globular and annular aggregates could be an experimental artifact due to instability of the tip-surface interaction during the image scan.

Moreover we emphasize that such soft aggregates were not modified by the repeated image scans when operating with a tip-sample force lower than 300pN.

Taken all together, these data indicate that the globular and annular structures visible in Fig. 6a, c are two distinct objects and are not a consequence of contrast inversion induced by instability of the tip-surface interaction. Accordingly, the structure at the upper right-hand corner of Fig. 6a is a globular peptide aggregate positioned immediately to the right of an underlying annular structure (enlarged in the insets to Fig. 6a) and similarly the aggregate in the centre of Fig. 6a is a globular oligomer nucleated on an annular structure partially exposed on the bilayer surface.

The imaging of the complex LA domains at the bottom right-hand corner of Fig. 6a and 6c shows that these annular aggregates favour the aggregation of further soft and less structured peptide material, forming a layer about 1.6 nm thick.

AFM images were also acquired using different tapping forces systematically varied within the range of 220-234pN. In Fig. 6g and 6h, globular aggregates composing the LA domains are imaged with an average force of 220pN and 234 pN, respectively. While the use of a 220pN tapping force does not induce any modification of the globular aggregate topography, a 234pN tapping force causes the complete removal of globular aggregates, with the simultaneous appearance of underlying annular structures. Accordingly with the previous observation (Fig. 6a, c), the globular aggregates appear positioned above the annular structures. As shown in Fig. 6h, defective mechanical stripping can occur when the tapping force is applied to both globular and annular structures: i) aggregates can be fragmented so that residual peptides can fill annular structures (large white ellipse in Fig. 6g and h where one annular structure is missing); and ii) annular structures are filled by peptide and, through mechanical stripping, are depleted (the small white ellipse highlights an annular structure filled in Fig. 6g that is depleted in Fig. 6h). From a statistical analysis of the radial cross-sections, annular structures with an average diameter of 20.0±0.5 nm protrude above the lipid surface by about 1 nm.

Finally Fig. 6i and 6l show high-resolution AFM images of LA on DOPC/DHA at t = 105 min and t = 140 min from Aβ(25-35) deposition. At 105 minutes (Fig. 6i), the peptide layer presents a linear re-organization of material as highlighted by fiber segment analysis (SPIP software). After 140 min, the LA domain is denser throughout, while linear organization is less visible though still distinguishable, as a consequence of both the growth process and sedimentation. In some locations, the LA is organized into annular structures that even though similar in dimension to the ones observed on DOPC (red circles in Fig. 6i) are more sporadic in number and of different nature.

## Discussion

The cell membrane defines a complex environment modulated by a plethora of chemical, physical and biological factors. Aβ peptide amyloidogenic aggregation is affected by the size, shape and relative content of molecules in such an environment. Indeed, the DHA molecule has recently been correlated with membrane fluidity and plasticity. In this work, AFM was used to visualize Aβ(25–35) aggregation on two surfaces with different fluidity, i.e. lipid DOPC and lipid DOPC/DHA bilayers. Experimental results are compared to the peptide grown on mica as reference surface.

Aβ(25–35) corresponds to the shortest fragment able to form large β-sheet aggregate and DOPC as well as DOPC/DHA bilayers are paradigm surfaces for mimicking cell-membrane environments with different fluidities in physiological solutions. As shown in the AFM measurements performed in PBS, in situ and in real time, peptide aggregation on DOPC and DOPC/DHA forms peptide layers driven by coalescence of globular aggregates nucleated on annular structures embedded in the lipid bilayers. DHA content affects the lipid environment, hence the peptide aggregation. Indeed, the growth of peptide layers on DOPC/DHA is faster than on DOPC. Both DOPC and DOPC/DHA show annular structures that, as a matter of principle, might be associated with oligomeric rings affecting the lipid membrane environment. This is a possible toxicity mechanism of β-amyloid peptide which damages the membrane by forming pores. [13]–[15]

Morphological analysis on AFM images indicates a mutual interaction between annular structures and lipid surfaces. On DOPC, LAs appear strongly bound to the bilayer which end-results into a membrane solubilization (also known as a detergent effect, Fig. 4). On DOPC/DHA bilayers, the growth kinetics of LAs is much faster than on DOPC (layer saturation is reached after only 140 minutes compared to 5 hours) and, lastly, the interaction among them gives rise to fibril-like superstructures (Fig. 6). Despite the same experimental conditions, DOPC/DHA solubilization is much less evident than in DOPC. Such differences for DOPC/DHA vs. DOPC, i.e. faster LA growth and lower detergent effect, may be due to the enhanced fluidity of the DOPC/DHA bilayer that promotes aggregate mobility, layer growth and reduced solubilization.

The increased fluidity of the bilayer containing DHA also reduces defects when it is formed on mica (Fig. 3). The morphological comparison of bilayers in PBS, without peptide addition, shows that DOPC/DHA is less defective (Θ_DOPC/DHA_ varies from 94 to 98%) than DOPC which has larger defects (Θ_DOPC_ from 88 to 96%). The pre-existence of defects may be considered an enhanced factor of bilayer sensitivity to the toxic action of the amyloid peptide. Indeed, regions where membranes are lacerated (defects) are micro-environments where LAs grow. In particular, defect edges, where phospholipids are poorly organized, are preferential sites for the detergent action of Aβ(25–35) whose amphipathic properties enable it to interact with the disordered phospholipids, thus enlarging the defects.

From the experimental results, the role of DHA in the bilayer is twofold:

- It protects the membrane from damage caused by interactions with peptide aggregates;
- It reduces the bilayer defects where the delipidation process starts.

Growing evidence has been accumulating to clarify the correlation between amyloid cascade and etiology of AD: i) amyloid peptide toxicity is prevalent when early oligomers affect neuronal membrane integrity and therefore neuronal functionality. In this case, amyloid plaques may be considered non toxic but neuroprotective [45]; ii) amyloid peptide toxicity is prevalent in conditions where neuronal cell compartments, including membranes, are already damaged. These conditions may be related to chronic inflammatory diseases, according to the known role of proinflammatory molecules, interleukin 1 (IL-1b) and TNFa converting enzyme, in the etiology of AD.

In this scenario, our data provide indications that a suitable amount of unsaturated DHA fatty acids could be the preliminary condition for preventing both chronic inflammatory processes and toxic actions of amyloid peptide and other similar neurodegenerative proteins.

## Supporting Information

S1 File **Figures S1-S3.** Figure S1: Representative force-distance approaching curve. Figure S2: Rupture force distributions for DOPC (A) and DOPC/DHA (B). Figure S3: Statistical distribution of Z_A-C_ and Z_B-C_ for DOPC.
(ZIP)
