# Supplementary figures and images for: Aggregation of Aß(25-35) on DOPC and DOPC/DHA Bilayers: An Atomic Force Microscopy Study
Source: PLoS One. 2014 Dec 31;9(12):e115780. doi: 10.1371/journal.pone.0115780 (PMC4281140; doi:10.1371/journal.pone.0115780)

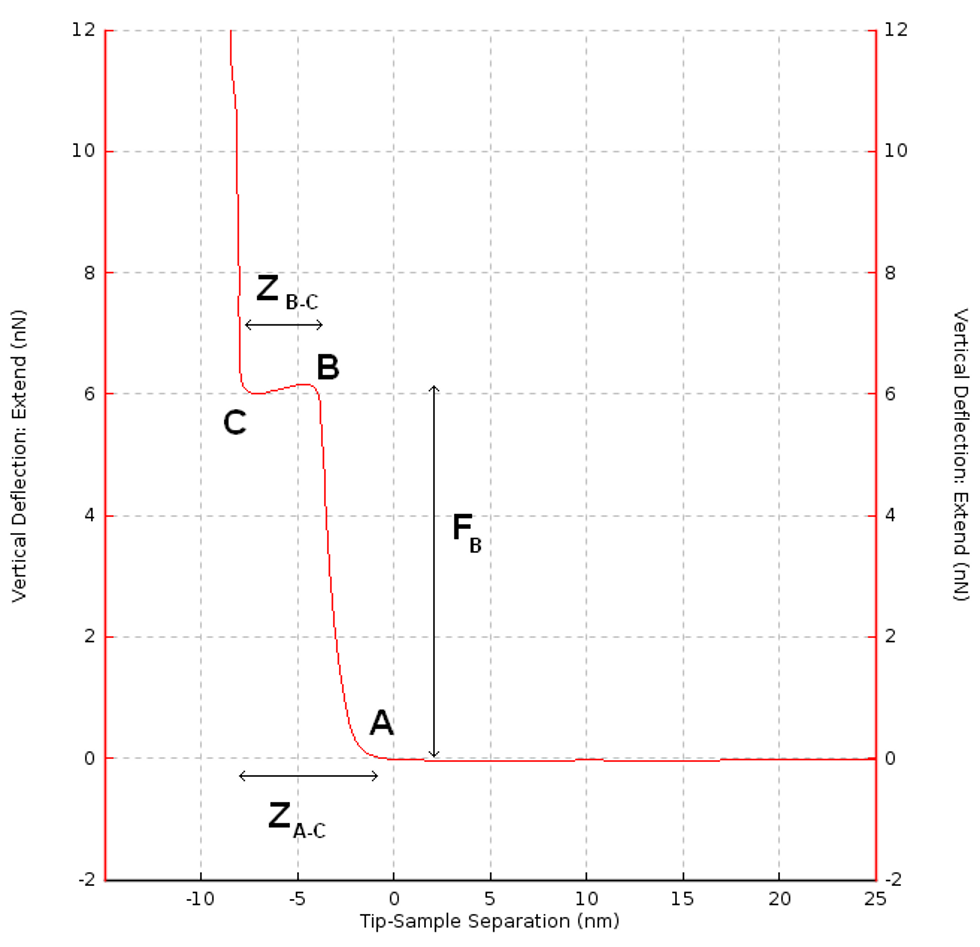

Supplement: S1 File — Figures S1-S3. Figure S1: Representative force-distance approaching curve. Figure S2: Rupture force distributions for DOPC (A) and DOPC/DHA (B). Figure S3: Statistical distribution of ZA-C and ZB-C for DOPC. (ZIP) [file pone.0115780.s001.zip › Figura S1.tif]

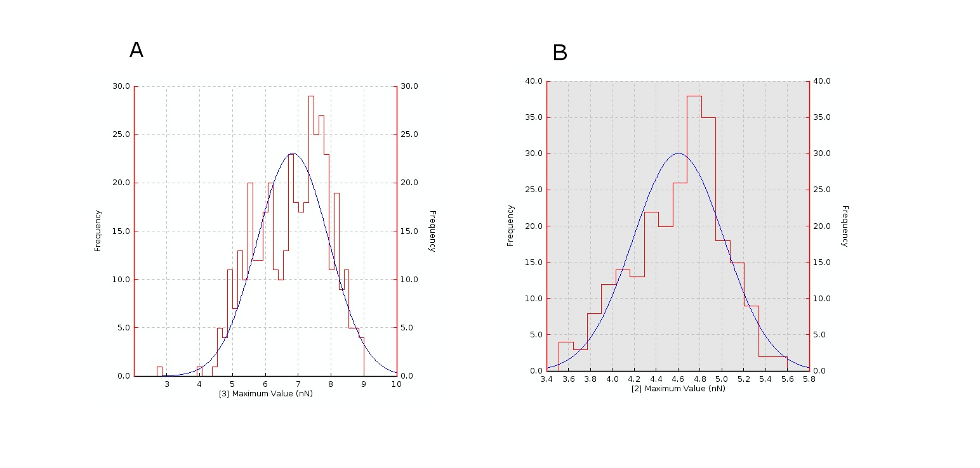

Supplement: S1 File — Figures S1-S3. Figure S1: Representative force-distance approaching curve. Figure S2: Rupture force distributions for DOPC (A) and DOPC/DHA (B). Figure S3: Statistical distribution of ZA-C and ZB-C for DOPC. (ZIP) [file pone.0115780.s001.zip › Figura S2.tif]

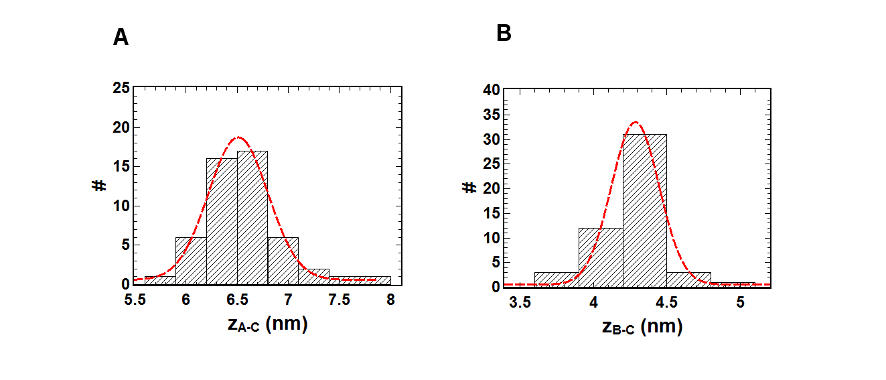

Supplement: S1 File — Figures S1-S3. Figure S1: Representative force-distance approaching curve. Figure S2: Rupture force distributions for DOPC (A) and DOPC/DHA (B). Figure S3: Statistical distribution of ZA-C and ZB-C for DOPC. (ZIP) [file pone.0115780.s001.zip › Figura S3.tif]
