# Supplementary material for: Aggregation of Aß(25-35) on DOPC and DOPC/DHA Bilayers: An Atomic Force Microscopy Study
Source: PLoS One. 2014 Dec 31;9(12):e115780. doi: 10.1371/journal.pone.0115780 (PMC4281140; doi:10.1371/journal.pone.0115780)
Supplement: S1 File — Figures S1-S3. Figure S1: Representative force-distance approaching curve. Figure S2: Rupture force distributions for DOPC (A) and DOPC/DHA (B). Figure S3: Statistical distribution of ZA-C and ZB-C for DOPC. (ZIP) [file pone.0115780.s001.zip › Supporting information_Dursi_4_11.doc]

**Supporting Information**

The thickness and rupture force of DOPC and DOPC/DHA bilayers were measured by statistically investigating force-distance curves. As shown in Figure S1, three characteristic points were defined in the approaching curve: **A** corresponds to the first contact point of the tip with the bilayer surface, **B** to the rupture of the bilayer through its thickness and **C** to the contact with the mica surface, respectively.

**Figure S1:** Representative force-distance approaching curve

As reported in ref. [1], the rupture force (F_B_) and the bilayer characteristic distances Z_A-C_, Z_B-C_ and Z_A-B_ were directly calculated from each curve.

For example, the distributions of the rupture forces for DOPC and DOPC/DHA, as extracted from 412 and 240 curves respectively, are shown in Fig. S2.

**Figure S2:** Rupture force distributions for DOPC (A) and DOPC/DHA (B)

From the statistical analysis, the mean value of F_B_ extracted from the Gaussian fit is (6.8±1.1)nN for DOPC and (4.6±0.4)nN for DOPC/DHA.

The same procedure was performed on each characteristic distance of the force-distance approaching curve. For example, the distributions of Z_A-C_ and Z_B-C_ for DOPC, as extracted from 50 curves, are reported in Fig. S3.

**Figure S3:** Statistical distribution of Z_A-C_ and Z_B-C_ for DOPC

For the sake of thoroughness, all Z values are commonly calculated (see Table T1) although the value most frequently used for bilayer thickness is Z_B-C_.

**TableT1: Z values for DOPC and DOPC/DHA**

|  | Z_A-C_ (nm) | Z_B-C_ (nm) | Z_A-B_ (nm) |
| --- | --- | --- | --- |
| DOPC | 6.5±0.6 | 4.3±0.3 | 2.3±0.6 |
| DOPC/DHA | 6.9±0.8 | 4.1±0.1 | 2.9±0.4 |

The average tip-sample force <F_ts_> applied to the sample from the tip was derived from equation 2.29 reported in the references [2]:

where A_0_ is the cantilever free amplitude, A_SP_ is the amplitude set point and F_0_=(k/Q)A_0_ depends on the elastic constant k and the quality factor Q of the cantilever. The free amplitude set point is measured from the microscope in mV and, through the deflection sensitivity α (expressed in nm/V), A_0_ and A_SP_ were converted into nm. The deflection sensitivity α and the elastic constant k were experimentally measured.

For example, the average force <F_ts_> used for imaging the sample described in Figure6g was calculated using the following values: A_0_=1000mV, A_SP_=800mV, k=0.6N/m, Q=20 and α=21.66nm/V. From them, F_0_=(k/Q)A_0_α is 727pN which is reduced to <F_ts_>=220pN for A_SP_/A_0_=0.8.

**References**

[1] SJ. Attwood, Y. Choi and Z. Leonenko, Preparation of DOPC and DPPC Supported Planar Lipid Bilayers for Atomic Force Microscopy and Atomic Force Spectroscopy, International journal of molecular sciences 14, 3514-3539 (2013)

[2] R. Garcia and R. Pérez, Dynamic atomic force microscopy methods, Surface Science Reports 47, 197-301 (2002)
